# Treatment of concomitant injuries of the DRUJ

**DOI:** 10.1186/1753-6561-9-S3-A36

**Published:** 2015-05-19

**Authors:** Jason Harvey

**Affiliations:** 1 Orthosport Victoria, Richmond, Victoria, 3121, Australia

## 

One of the more difficult problems in wrist surgery is an injury to the ulna side of the wrist associated with a distal radius fracture. When should these be treated acutely and when do they resolve without the need for surgical intervention? The long term disability due to DRUJ problems is significant and includes pain, instability, loss of motion or any combination of the above.

Importantly not all patients distal radioulnar joints have the same amount of “play” and therefore assessment of the contralateral side pre operatively (if uninjured) is beneficial prior to surgical treatment of a distal radius fracture to help guide the decision making process. Geissler et al in 1996 classified these injuries into:

Type I – Stable Distal Radioulnar Joint Lesions

Type II – Unstable Distal Radioulnar Joint Lesions

Type III – Potentially Unstable Distal Radioulnar Joint Lesions

Injuries can be to the soft tissues, the bone or both.

## Distal radius

Inadequate reduction of the distal radius can lead to instability on the ulna side of the wrist. The main problems are:

1/ Inadequate reduction of the sigmoid notch, especially the dorsoulnar fragment

2/ Radial translation of the distal fragment leading to “detensioning” of the interosseous membrane and consequent DRUJ instability

3/ Inadequate restoration of the volar tilt of the radial articular surface

If these factors have been addressed then attention should focus on the ulna sided structures and the role they have in maintaining stability of the wrist

## Bony injuries

Fracture of the distal ulna frequently occurs with a fracture of the distal radius, most commonly involving the ulna styloid. Less common is instability of the DRUJ associated with this. Predicting which fractures lead to instability can be difficult. It is impractical to get an MRI on every patient to evaluate the integrity of the TFCC and instability is not necessarily a consequence of a TFCC tear, therefore guidelines can be developed for assessing and treating styloid fractures.

1/ Small tip fractures & through the mid styloid do not often lead to instability, no fixation required

2/ Larger fragments at the base of the styloid are more likely to lead to instability as they may damage the foveal fibres, consider fixation, especially if displaced

3/ Fracture of the head/neck of the ulna should be treated on the basis of stability and involvement of the articular surface with restoration of joint congruity the main aim.

Whether or not to stabilise base of styloid fractures leads to heated debate among many surgeons with proponents of both approaches.

## Soft tissue injury (TFCC)

Palmer has classified traumatic injuries to the TFCC and these are the most common soft tissue injury to the ulna side of the wrist that can affect the DRUJ and its stability

Class I are traumatic tears of the triangular fibrocartilage complex according to his classification.

**Type A** have a central perforation

**Type B** involve peripheral ulnar tears

**Type C** are distal tears

**Type D** are radial tears of the triangular fibrocartilage complex from the radius.

Guidelines to treatment are as follows:

1. Evaluation of DRUJ stability after stabilisation of the distal radius fracture

2. If there is no instability – treat the DRUJ as for the distal radius fracture

3. If instability is present, evaluate if stable in pronation or supination

4. If it is stable then immobilise in the position of stability for 4 weeks before slowly regaining motion

5. If there is no position of stability – arthroscopy and repair TFCC tear as needed

6. If there is no tear to repair and remains unstable then pin the joint with a 2mm K-wire, proximal to the joint.

7. If no arthroscopy is available, pin the DRUJ with a 2mm K-wire just proximal to the joint to minimise articular damage.

**Figure 1:**
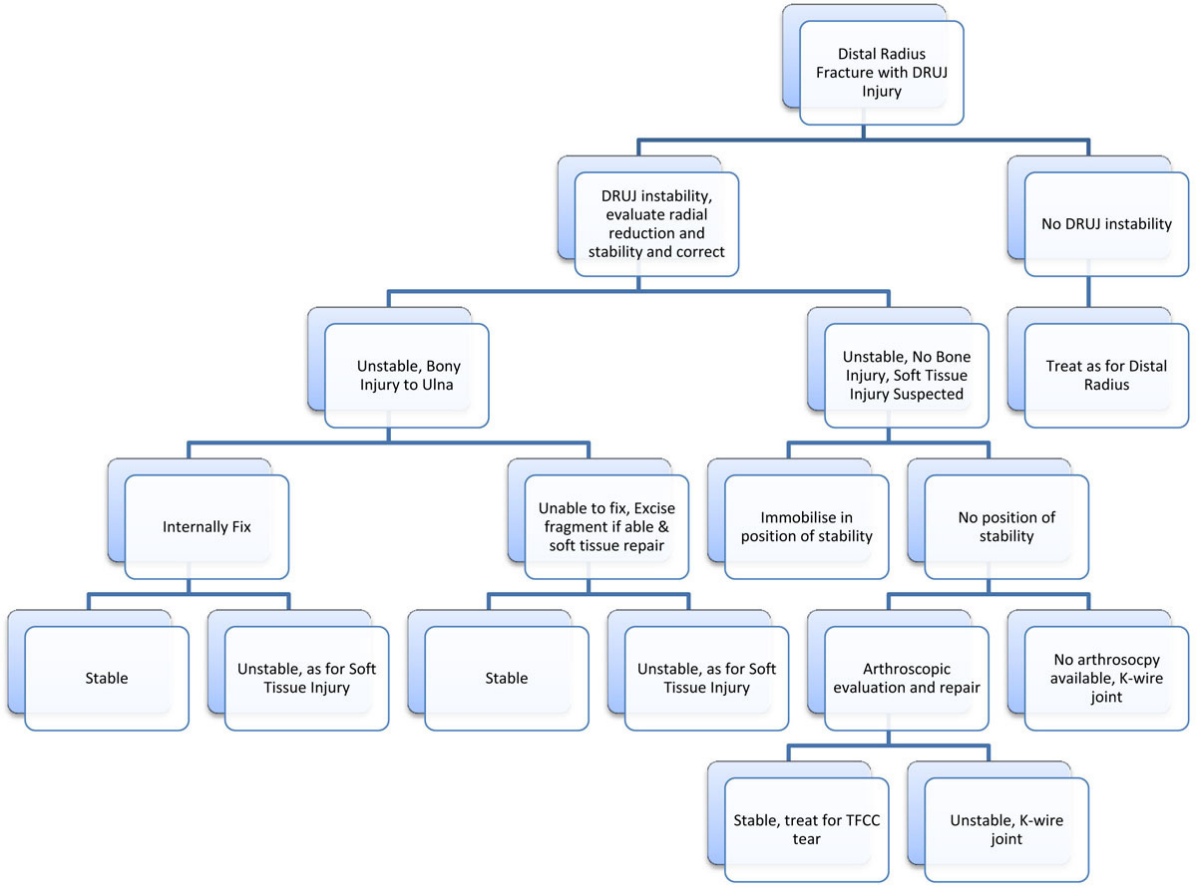
In flow chart form

